# The Use and Outcomes of Motor Rehabilitation Services Among People With Cerebral Palsy Change Across the Lifespan

**DOI:** 10.3389/fneur.2021.771348

**Published:** 2022-02-10

**Authors:** Gwenaël Cornec, Sylvain Brochard, Gaelle Drewnowski, Isabelle Desguerre, Philippe Toullet, Audrey Fontaine, Yann Le Lay, Julia Boivin, Eric Bérard, Maria Bodoria, Vincent Gautheron, Javier De la Cruz

**Affiliations:** ^1^Physical and Rehabilitation Medicine Department, University Hospital of Brest, Brest, France; ^2^Medical Research and Training Unit, Western Brittany University, Brest, France; ^3^Pediatric Physical and Rehabilitation Medicine Department, Fondation Ildys, Brest, France; ^4^Institute of Health and Medical Research (INSERM) UMR 1101, Medical Data Treatment (LaTIM), Brest, France; ^5^Expert Patient, Lyon, France; ^6^Hôpital Necker – Enfants malades, Pediatrics-Radiology-Genetics, Paris, France; ^7^Institut Motricité Cérébrale – Cercle de Documentation et d'Information pour la rééducation des Infirmes Moteurs Cérébraux, Paris, France; ^8^“A Pas de Géants”, Paris, France; ^9^ISIR, UMR 7222 CNRS, Agathe Group INSERM U 1150, Sorbonne University, Paris, France; ^10^Private Practice, Nantes, France; ^11^IFM3R Institut Régional de Formation aux Métiers de la Rééducation et de la Réadaptation des Pays de la Loire, Nantes, France; ^12^Odyneo, Lyon, France; ^13^Fondation Paralysie Cérébrale, Paris, France; ^14^Department of Pediatric Physical and Rehabilitation Medecine, CHU Bellevue - Saint-Etienne, LIBM, Université Jean Monnet Saint Etienne et Université de Lyon, Saint-Étienne, France; ^15^Hospital Universitario 12 Octubre, Madrid, Spain; ^16^Health Research Institute Imas12, Madrid, Spain; ^17^Mother & Child Health and Development Network (SAMID Network), National Health Institute (ISCIII), Madrid, Spain

**Keywords:** disability, cerebral palsy, rehabilitation, healthcare service, transition to adult care, adult neurology

## Abstract

**Background and Aims:**

The provision of coordinated and multidisciplinary rehabilitation programs that adapt to the individual with cerebral palsy (CP) evolving rehabilitation needs throughout the different phases of life is highly challenging for healthcare systems. The aim of this study was to report the changes in motor rehabilitation (MR) environmental factors, service use and patient outcomes between children and adults with cerebral palsy and to identify if changes took place earlier or later than the standard division between pediatric and adult healthcare systems at 18 years.

**Methods:**

We used data from the French ESPaCe survey to select a set of indicators for MR environmental factors, service use and patient outcomes, highlighted by patients and families in previous studies. We then compared the distribution of the indicator data between children and adults, as well as between four transition age groups: children under 12, adolescents up to 17 years, young adults, and adults over 25 years of age. We estimated odds ratios adjusted for motor involvement, associated impairments and informant type.

**Results:**

A total of 997 respondents over 2 years of age were included in this study (484 children and 513 adults). Finding an available physiotherapist was very difficult for almost half of the children, and a greater proportion of adolescents and adults. Physiotherapy was provided in a private outpatient practice for twice as many adults over 25 years as children and adolescents. The weekly amount of physical therapy decreased as outpatient practice increased. Multidisciplinary rehabilitation decreased sharply from adolescence and was halved at adulthood. Satisfaction with the MR program decreased from childhood into adolescence and adulthood. Perceived impact of physiotherapy on people with CP and their main carers were less positive in adolescents.

**Conclusions:**

Healthcare policies should focus on accessibility issues at all ages, consider adolescents as a specific population, consider a wide transition phase (12–25 yo) and maintain a multidisciplinary approach at adulthood. There is a strong need for national rehabilitation strategies for individuals with CP.

## Introduction

Coordinated and multidisciplinary rehabilitation is essential to fully address the health problems encountered by individuals with cerebral palsy (CP) over their lifetime. Despite the fact CP is a lifelong condition, most research on rehabilitation for people with CP has been conducted in children (1). Although the cerebral lesions that interfere with the brain development are non-progressive, deteriorations occur early in all dimensions of the International Classification of Functioning with aging. In particular, mobility becomes increasingly limited, pain increases and cardio-vascular diseases and cognitive disorders develop earlier than expected (2–12). These changes in health status over the course of a person's life generate different medical and rehabilitation needs, which have recently become the object of a growing research interest in adults with CP. However, the provision of coordinated and multidisciplinary rehabilitation programs that adapt to each individual's evolving rehabilitation needs throughout the different phases of life is highly challenging for healthcare systems.

Pediatric and adult healthcare services are quite distinct and the transition to adult services is rarely smooth; people with CP often report experiencing a void when they leave the pediatric system (13–15). Although clinical guidelines for the childhood-adulthood transition have been established in different countries over the past 10 years (16, 17), young adults with CP continue to report that the transition between life phases is problematic (18). In France, the healthcare system provides 100% coverage of all healthcare expenditures under a national solidarity scheme (“Assurance Maladie – Sécurité Sociale”) to thirty chronic conditions, including CP, whereas other conditions receive 65–80% coverage. This extended financial coverage does not seem to avoid difficulties for people with CP in the transition phase. For instance, a regional study of 502 individuals with CP found that the use of medication increased with age, however the provision of physical types of health care (rehabilitation, physical medicine and rehabilitation follow-up, and provision of equipment) decreased, independently of ambulatory status. The drop in service provision occurred mainly after the transition to adult services (19). Individuals with CP frequently report that the transition between healthcare systems is a “brutal” experience. Furthermore, the transition to adult services occurs at a fixed age, which does not necessarily correspond to the individual's needs. It is highly likely that the period of transition actually starts during adolescence (≥12 yo) and ends in the late twenties (20). Indicators need to be determined so that transition between rehabilitation services can be tailored appropriately. To ensure the relevance of such evidence-based adaptations, public views must be taken into account to prioritize actions, establish the importance of specific outcomes and generate patient preference-informed guidelines (21).

The ESPaCe survey was a national survey designed to report the unmet needs and expectations about motor rehabilitation (MR) of children, adolescents and adults with CP and their families in France (22, 23). The questionnaire was co-designed by service users and professionals to evaluate chosen key indicators of the health care user's experience such as self-reported environmental factors (access to rehabilitation, MR coordination, rehabilitation settings etc.), rehabilitation service use (amount of physiotherapy, multidisciplinary teams etc.) and patient outcomes and experiences (satisfaction, impact on activities of daily living etc.). Evaluating the changes throughout the lifespan of such modifiable, self-reported factors would guide the national development of rehabilitation services that consider the different phases of an individual's life.

We hypothesized that MR environmental factors, service use and patient outcomes and experiences reported as indicators by people with CP would change between childhood and adulthood, and that some changes would occur during adolescence (12–17 yo) and others would occur in young adults (18–25 yo) or later in life, depending on the indicators.

The aim of this study was to report the changes in motor rehabilitation (MR) environmental factors, service use and patient outcomes between children and adults with CP and to identify if changes took place earlier or later than the standard division between pediatric and adult healthcare systems at 18 years.

## Methods

### Participants

The Enquête Satisfaction Paralysie Cérébrale (ESPaCe: cerebral palsy satisfaction survey) was a cross-sectional study coordinated by a CP research foundation (Fondation Paralysie Cérébrale, France) in collaboration with patient and professional organizations (22). People were included if they reported living in France with a motor impairment consistent with the definition of CP (24). They were excluded if the descriptive information provided in the survey regarding their motor impairment was insufficiently detailed or not consistent with the definition of CP (e.g., progressive disorders). The present study included respondents who were at least 2 years of age and both those who were undergoing MR and those who were not.

The study fulfilled the French legal data protection requirements at the time of the data collection. The ESPaCe survey was registered in ClinicalTrials.gov with the identifier NCT04509544.

### Study Variables

The ESPaCe questionnaire was developed by a multidisciplinary group that included individuals with CP and representatives from patient and family organizations, professional and scientific societies. The questions covered the topics identified in a preliminary qualitative study that involved in-depth interviews with individuals with CP and their families.

#### Outcomes

For this study, we selected a set of questionnaire items prioritized by ESPaCe participants as indicators of MR environment, service use and patient outcomes.

##### MR Environmental Factors

Participants were asked about the availability of physiotherapists, the access to a physiotherapist trained in CP rehabilitation, the care setting in which they attended MR (private outpatient clinic vs. healthcare organization), the presence of an identified healthcare professional coordinating their MR and of regular communication between professionals.

##### Rehabilitation Service Use

Participants reported their current participation in MR, the weekly amount of physical therapy (PT) received (≥90 min per week), MR multidisciplinarity (two or more therapies) and whether the goal setting process was shared.

##### Rehabilitation Service Patient Outcomes

Satisfaction with rehabilitation services was evaluated using the CSQ-8 questionnaire (25), satisfaction with pain management during PT sessions, perceived outcome of MR (impact of MR on activities of daily living and quality of life for people with CP and for their main carer).

Some outcome responses were dichotomous (service provider, MR multidisciplinarity, attending school or work) but most were scales 0–5 (availability of physiotherapists, access to a physiotherapist with specific training, communication between professionals, satisfaction with pain management, shared physiotherapy goal setting) or −5 to 5 (impact of MR on people with CP and their main carer).

#### Main Determinant

Participant age was the main study determinant. Age was dichotomized at 18 years to mirror the split between pediatric and adult healthcare systems. To further explore the transition age, the variable was categorized in four levels: children (2–11 years), adolescents (12–17 years), young adults (18–24 years) and over 25 years old.

#### Population Factors

Participants reported their gender, CP subtype, Gross Motor Function Classification System (GMFCS) and Manual Ability Classification System (MACS) levels, associated impairments (severe visual, hearing, intellectual impairment, and epilepsy), mother education, frequency of episodes of pain, participation in school or professional activities.

The questionnaire was released in both web and paper format. Participation was open from June 2016 to June 2017. The study was promoted nationally through advocacy groups, scientific and professional societies and social media, and locally by patient associations and healthcare professionals. The questionnaire instructions stated that it should be preferably self-reported by the individual with CP or proxy-reported by the main carer, and that professionals involved in MR should not be asked to help complete the questionnaire.

### Statistical Analysis

The distribution of population factors and outcome variables were described across the 2 and 4 age groups. For clarity of presentation of the univariate results, outcome variables measured on scales were dichotomized. Proportions were compared across age groups using a Cochran-Armitage trend test, Chi-square test or Kruskal-Wallis test. The adjusted age effects were estimated for the 2 and 4 age category variables. The dependent variables were analyzed as ordinal with proportional odds logistic regression models after checking the proportional odds assumption, or with binary logistic models. Multivariable age effect estimates were adjusted on gender, CP subtype (unilateral or bilateral spastic CP vs. dyskinetic/ataxic CP), GMFCS, visual or hearing impairments (severe), intellectual impairment (severe, moderate, mild/no), epilepsy, and informant type. Odds ratios and 95% confidence intervals (95% CI) are presented for the 2 and 4 age category variables. In addition, if no differences between the 4 age categories were identified, a reduced age variable was fitted in the model and estimates reported if the predictive ability of the model improved and new age category differences showed a *p*-value < 0.1. The complete record analysis was implemented under the assumption that data missingness was not related to both the main predictor (age) and the outcomes. Sensitivity analyses were performed to assess the impact in the results of rehabilitation non-users and of the choice of adjustment variables – e.g., socioeconomic indicator. The analysis for this paper was generated using SAS/STAT software version 9.4/14.2 (SAS Institute Inc., Cary, NC, USA).

## Results

### Study Population

Out of 1,010 eligible participants in the ESPaCe survey, 997 over the age of 2 years were included in the present study: 341 (34%) were children (2–11 yo), 143 (15%) were adolescents (12–17 yo), 111 (11%) were adults aged between 18 and 25 yo and 398 (40%) were older than 25 yo; 54% were male.

The CP subtypes reported were unilateral spastic (32%), bilateral spastic (54%), dyskinetic (11%) and ataxic (4%). Thirty-three percent of respondents had a Gross Motor Function Classification System (GMFCS) level of I-II, 19% had level III, and 47% had levels IV-V. Fifty-one percent reported at least one severe associated impairment (intellectual, visual, auditory or epilepsy). Table 1 shows the differences in individual characteristics by age group. The proportion of participants with at least one severe impairment was lower in children (44%) than in adolescents or adults (54%), *p* < 0.004. The proportion of mothers with higher education decreased in successive age groups, from 80% in children to 44% in adults, *p* < 0.001. Frequent episodes of pain were reported by 9% of children and 35% of adults over 25 yo, *p* < 0.001. Participation in school or employment decreased in successive age groups, from 90% in children to 26% after 25 years, *p* < 0.001.

**Table 1:** Age distribution of population factors as reported in ESPaCe, the French National Survey on Motor Rehabilitation Services.

**Population factors**			**Age distribution (Transition age groups)**					
		**Missing data**	**02–11 y**	**12–17 y**	**18–24 y**	**25–74 y**	**Total**	
		***N*** **=** **997**	***N*** **=** **341**	***N*** **=** **143**	***N*** **=** **111**	***N*** **=** **398**	***N*** **=** **997**	***p*** **-value**
	**Response categories**	**%**	***n*** **(%)**	***n*** **(%)**	***n*** **(%)**	***n*** **(%)**	**%**	
**Non-modifiable factors**								
**Gender**		1%						0.003
	Male		201 (59)	84 (59)	48 (44)	195 (50)	528 (54)	
CP Subtype		10%						<0.0001
	Unilateral spastic CP		136 (43)	42 (33)	21 (21)	90 (26)	289 (32)	
	Bilateral spastic CP		139 (44)	70 (54)	66 (65)	208 (59)	483 (54)	
	Dyskinetic CP		34 (11)	11 (9)	10 (10)	41 (12)	96 (11)	
	Ataxic CP		9 (3)	6 (5)	4 (4)	14 (4)	33 (4)	
Gross motor function classification system		5%						<0.0001
	Level I		67 (21)	19 (14)	11 (10)	19 (5)	289 (12)	
	Level II		93 (29)	29 (21)	25 (23)	56 (15)	203 (21)	
	Level III		40 (12)	29 (21)	15 (14)	93 (25)	177 (19)	
	Level IV		56 (17)	26 (19)	27 (25)	110 (29)	219 (23)	
	Level V		69 (21)	33 (24)	29 (27)	99 (26)	230 (24)	
Manual ability classification system		7%						0.3
	Level I		36 (11)	14 (10)	7 (7)	55 (15)	112 (12)	
	Level II		121 (38)	40 (29)	47 (44)	126 (34)	334 (36)	
	Level III		83 (26)	28 (21)	23 (22)	75 (20)	209 (22)	
	Level IV		40 (12)	29 (21)	14 (13)	57 (16)	140 (15)	
	Level V		41 (13)	25 (18)	15 (14)	54 (15)	135 (15)	
Associated impairments								
	Severe visual imp.	7%	40 (14)	18 (13)	18 (18)	51 (14)	127 (14)	0.4
	Severe hearing imp.	7%	6 (2)	8 (6)	5 (5)	11 (3)	30 (3)	0.5
	Severe intellectual imp.	14%	40 (14)	26 (20)	22 (22)	68 (20)	156 (18)	0.5
	Epilepsy	9%	88 (28)	41 (32)	36 (37)	120 (34)	285 (32)	0.073
Mother education		24%						<0.0001
	Higher education		226 (80)	84 (77)	57 (64)	118 (44)	485 (65)	
**Modifiable factors**								
Pain, frequency (0–5)		21%						<0.0001
	0 - No episodes		88 (31)	24 (24)	19 (22)	49 (16)	180 (23)	
	4–5 - High frequency		26 (9)	19 (19)	24 (28)	109 (35)	178 (23)	
Schooling or professional activities		3%						<0.0001
	Involved		298 (90)	106 (77)	46 (42)	100 (26)	550 (57)	
**Informant**								
Questionnaire respondent		2%						<0.0001
	Family members		335 (100)	111 (80)	69 (63)	175 (45)	690 (71)	
	Individuals with CP		0%	28 (28)	41 (37)	212 (55)	281 (29)	

**Figure 1:**
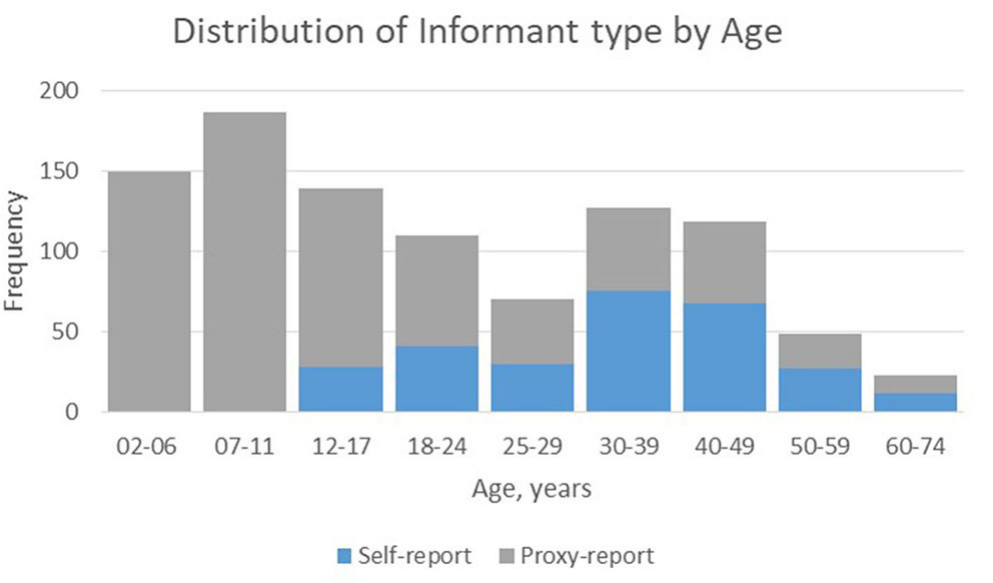
Informant type: self-reported vs. proxy-reported by age.

Seventy-one percent of questionnaires were answered by a family member. Twenty percent of the 12–17 yo, 37% of the 18–24 yo and 55% of the ≥25 yo completed the questionnaire themselves. Participants included in the study lived in twelve of the thirteen regions of France. No responses were received from the least populated region; participation from the most populated region (21%) was proportional to its relative population at the national level (19%) (Figure 1).

Data missingness on population factors was lower than 10% except for intellectual impairment (14%) and mother's education (24%), see Table 1. Missing data on outcomes was mostly in the range of 20–25%, see Table 2.

### Motor Rehabilitation Environmental Factors

**Figure 2:**
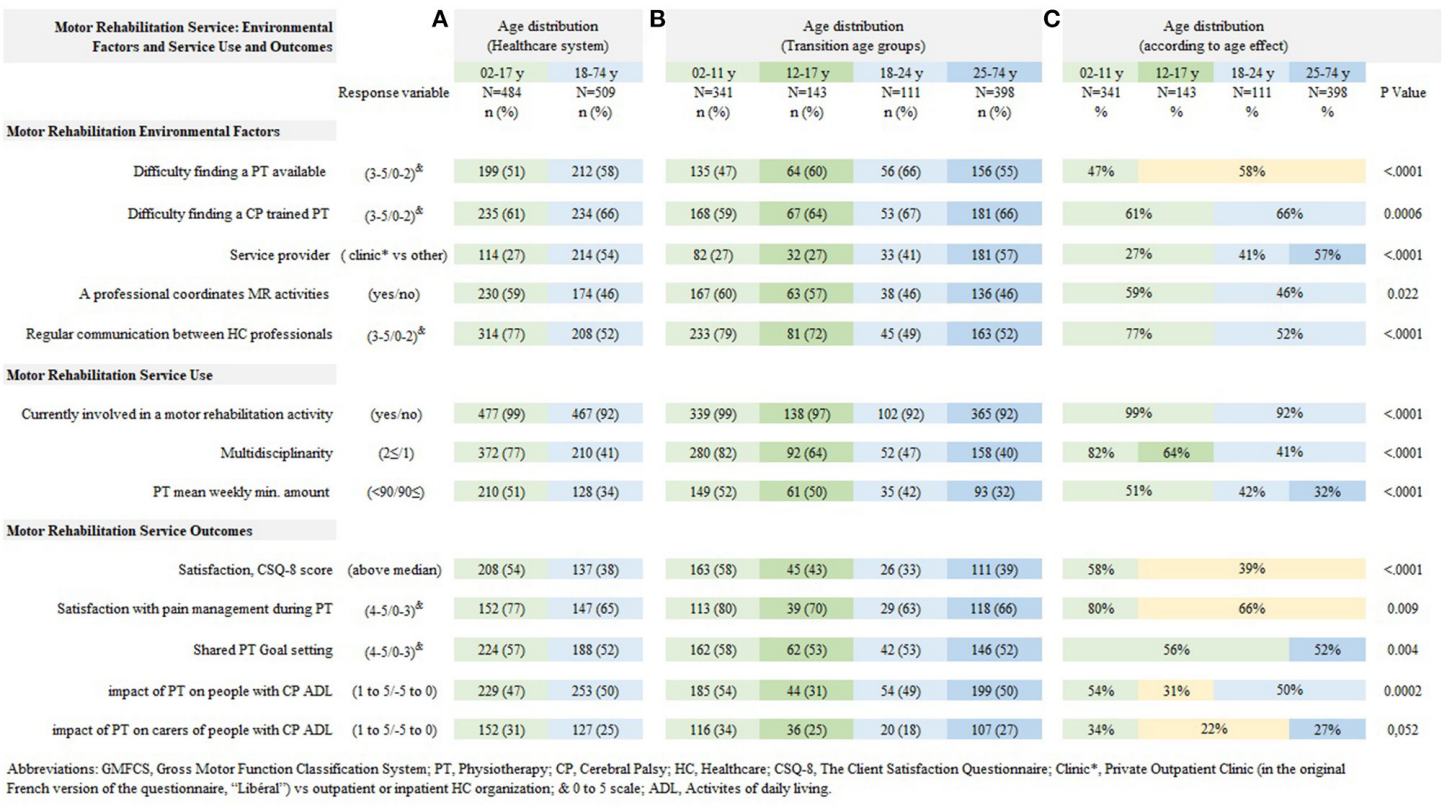
Age distribution of Motor Rehabilitation Service Factors. Age categories according to **(A)** healthcare system (adult and pediatric), **(B)** transition age groups, and **(C)** according to adjusted age effect on multivariable analysis.

Finding an available physiotherapist was reported as very difficult for 47% of children, and even more for adolescents and adults, 58%, odds ratio 2.3 (1.6–3.4). Finding a physiotherapist trained in CP rehabilitation was reported as very difficult for 61% of children and adolescents and 66% of adults, odds ratio 2.0 (2.3–3.0). Physiotherapy was provided in a private outpatient practice more frequently in young adults than adolescents, 27 vs. 41%, odds ratio 2.7 (1.2–6.0), and even more in adults over 25 years, 57%, odds ratio 2.1 (1.1–4.1). The presence of an MR coordinator was less frequently reported for adults than for children and adolescents, 46 vs. 59%, odds ratio 0.59 (0.38–0.93). Regular communication between health professionals was less frequently reported in adults, 52 vs. 77%, odds ratio 0.38 (0.27–0.56). Table 2 shows the age effects on multivariable analysis: Table 2A, shows the adult vs. pediatric age effect; Table 2B shows the transition age effects. Figure 2 summarizes the age distribution of the service indicators according the health system split, the transition age categories and according to the age effect.

**TABLE 2A:** Age effect on motor rehabilitation service factors adjusted on GMFCS, CP subtype, severe visual, hearing and intelligence impairments, epilepsy, gender and informant type with binomial/ordinal logistic regression models.

**Motor rehabilitation service: environmental factors, service use and outcomes**			**Adult vs. pediatric age effect**				
			**A**				
	**Missing data** ***N*** **=** **997**	**Response variable**		**Odds ratio estimate**			***p*** **-value**
			**Reference category**	**Point estimate**	**(95% CI)**		
**Motor rehabilitation environmental factors**							
Difficulty finding a PT available	24%	Ordinal (0–5)	18–74 vs. 02–17 y	1.8	1.2	2.7	0.003
Difficulty finding a CP trained PT	26%	Ordinal (0–5)	18–74 vs. 02–17 y	2.0	1.3	3.0	0.0006
Service provider^*^	18%	Binary	18–74 vs. 02–17 y	4.8	2.9	8.1	<0.0001
A professional coordinates MR activities	23%	Binary (yes/no)	18–74 vs. 02–17 y	0.59	0.38	0.93	0.022
Regular communication between HC professionals	18%	Ordinal (0–5)	18–74 vs. 02–17 y	0.38	0.27	0.56	<0.0001
**Motor rehabilitation service use**							
Currently involved in a motor rehabilitation activity	0%	Binary (yes/no)	18–74 vs. 02–17 y	0.21	0.096	0.45	<0.0001
PT mean weekly min. amount	21%	Binary (<90/90 ≤ )	18–74 vs. 02–17 y	0.30	0.19	0.47	<0.0001
Multidisciplinarity	0%	Binary (2 ≤ /1)	18–74 vs. 02–17 y	0.25	0.16	0.37	<0.0001
**Motor rehabilitation service outcomes**							
Satisfaction, CSQ-8 score	25%	Ordinal (quartiles)	18–74 vs. 02–17 y	0.47	0.32	0.71	0.0003
Satisfaction with pain management during PT^&^	30%	Ordinal (0–5)	18–74 vs. 02–17 y	0.59	0.36	0.95	0.031
Shared PT Goal setting	24%	Ordinal (0–5)	18–74 vs. 02–17 y	0.66	0.45	0.97	0.035
Impact of PT on people with CP ADL^#^	23%	Binary (1 to 5/−5 to 0)	18–74 vs. 02–17 y	1.3	0.89	1.9	0.18
Impact of PT on carers of people with CP ADL^#^	30%	Binary (1 to 5/−5 to 0)	18–74 vs. 02–17 y	0.89	0.59	1.3	0.58

*Age: 2 categories, 18–74 vs. 02–18 y*.

GMFCS, Gross Motor Function Classification System; PT, Physiotherapy; CP, Cerebral Palsy; HC, Healthcare; CSQ-8, The Client Satisfaction Questionnaire; Clinic^*^, Private Outpatient Clinic (in the original French version of the questionnaire, “Libéral”) vs. outpatient or inpatient HC organization; ^&^0–5 scale; ^#^ADL, Activites of daily living.

**TABLE 2B:** Age effect on motor rehabilitation service factors adjusted on GMFCS, CP subtype, severe visual, hearing and intelligence impairments, epilepsy, gender and informant type with binomial/ordinal logistic regression models.

**Motor rehabilitation service: environmental factors, service use and outcomes**			**Transition age effect**					
			**B**		
	**Missing data** ***N*** **=** **997**	**Response variable**		**Odds ratio estimate**			***p*** **-value**	
			**Reference category**	**Point estimate**	**(95%CI)**		**Category**	**Overall**
**Motor rehabilitation environmental factors**								
Difficulty finding a PT available	24%	Ordinal (0–5)	12–17 vs. 02–11 y	2.0	1.2	3.3	0.004	0.0004
			18–24 vs. 12–17 y	1.5	0.82	2.7	0.19	
			25–74 vs. 18–24 y	0.75	0.44	1.3	0.29	
			12–74 vs. 02–11 y	2.3	1.5	3.4	<0.0001	
Difficulty finding a CP trained PT	26%	Ordinal (0–5)	12–17 vs. 02–11 y	1.4	0.84	2.2	0.20	0.003
			18–24 vs. 12–17 y	1.4	0.76	2.6	0.27	
			25–74 vs. 18–24 y	1.3	0.74	2.2	0.37	
Service provider^*^	18%	Binary	12–17 vs. 02–11 y	1.1	0.58	2.1	0.78	<0.0001
			18–24 vs. 12–17 y	2.7	1.2	6.0	0.013	
			25–74 vs. 18–24 y	2.1	1.1	4.1	0.034	
A professional coordinates MR activities	23%	Binary (Yes/No)	12–17 vs. 02–11 y	0.82	0.47	1.4	0.48	0.021
			18–24 vs. 12–17 y	0.71	0.35	1.4	0.34	
			25–74 vs. 18–24 y	0.91	0.49	1.7	0.76	
Regular communication between HC professionals	18%	Ordinal (0–5)	12–17 vs. 02–11 y	0.79	0.49	1.3	0.3	<0.0001
			18–24 vs. 12–17 y	0.48	0.27	0.85	0.013	
			25–74 vs. 18–24 y	0.90	0.55	1.5	0.7	
**Motor rehabilitation service use**								
Currently involved in a motor rehabilitation activity	0%	Binary (yes/no)	12–17 vs. 02–11 y	1.2	0.35	3.9	0.81	<0.0001
			18–24 vs. 12–17 y	0.16	0.05	0.54	0.003	
			25–74 vs. 18–24 y	1.3	0.59	2.8	0.52	
PT mean weekly min. amount	21%	Binary (<90/90 ≤ )	12–17 vs. 02–11 y	0.81	0.47	1.4	0.45	<0.0001
			18–24 vs. 12–17 y	0.57	0.28	1.2	0.12	
			25–74 vs. 18–24 y	0.47	0.25	0.89	0.020	
			18–24 vs. 02–17 y	0.51	0.27	0.96	0.037	<0.0001
			25–74 vs. 18–24 y	0.47	0.25	0.90	0.022	
Multidisciplinarity	0%	Binary (2 ≤ /1)	12–17 vs. 02–11 y	0.31	0.18	0.53	<0.0001	<0.0001
			18–24 vs. 12–17 y	0.59	0.32	1.1	0.099	
			25–74 vs. 18–24 y	0.67	0.39	1.2	0.15	
			12–17 vs. 02–11 y	0.32	0.19	0.54	<0.0001	<0.0001
			18–74 vs. 12–17 y	0.44	0.27	0.72	0.0009	
**Motor rehabilitation service outcomes**								
Satisfaction, CSQ-8 score	25%	Ordinal (quartiles)	12–17 vs. 02–11 y	0.45	0.28	0.73	0.001	<0.0001
			18–24 vs. 12–17 y	0.71	0.37	1.3	0.29	
			25–74 vs. 18–24 y	1.4	0.74	2.7	0.92	
			12–74 vs. 02–11 y	0.38	0.25	0.57	<0.0001	
Satisfaction with pain management during PT^&^	30%	Ordinal (0–5)	12–17 vs. 02–11 y	0.59	0.32	1.1	0.088	0.050
			18–24 vs. 12–17 y	0.93	0.43	2.0	0.86	
			25–74 vs. 18–24 y	0.83	0.44	1.6	0.55	
			12–74 vs. 02–11 y	0.52	0.32	0.85	0.009	
Shared PT goal setting^&^	24%	Ordinal (0–5)	12–17 vs. 02–11 y	0.92	0.58	1.5	0.72	0.036
			18–24 vs. 12–17 y	1.0	0.56	1.9	0.90	
			25–74 vs. 18–24 y	0.57	0.33	0.99	0.047	
			25–74 vs. 2–24 y	0.57	0.39	0.83	0.004	
	**Missing data** ***N*** **=** **997**	**Response variable**		**Odds ratio estimate**			***p*** **-value**	
			**Reference category**	**Point estimate**	**(95%CI)**		**Category**	**Overall**
**Motor rehabilitation service outcomes**								
Impact of PT on people with CP ADL^#^	23%	Binary (1 to 5/−5 to 0)	12–17 vs. 02–11 y	0.38	0.23	0.61	<0.0001	0.0008
			18–24 vs. 12–17 y	2.3	1.3	4.3	0.007	
			25–74 vs. 18–24 y	0.97	0.57	1.6	0.91	
			02–11 vs. 12–17 y	2.7	1.6	4.4	<0.0001	0.0002
			18–74 vs. 12–17 y	2.3	1.4	3.7	0.0008	
Impact of PT on carers of people with CP ADL^#^	30%	Binary (1 to 5/−5 to 0)	12–17 vs. 02–11 y	0.70	0.42	1.2	0.18	0.071
			18–24 vs. 12–17 y	0.65	0.31	1.3	0.23	
			25–74 vs. 18–24 y	2.0	1.1	3.8	0.029	
			12–24 vs. 02–11 y	0.60	0.38	0.96	0.032	0.052
			25–74 vs. 12–24 y	1.6	1	2.5	0.0499	

*Age: 4 transition categories*.

GMFCS, Gross Motor Function Classification System; PT, Physiotherapy; CP, Cerebral Palsy; HC, Healthcare; CSQ-8, The Client Satisfaction Questionnaire; Clinic^*^, Private Outpatient Clinic (in the original French version of the questionnaire, “Libéral”) vs. outpatient or inpatient HC organization; ^&^0–5 scale; ^#^ADL, Activites of daily living.

### Motor Rehabilitation Service Use

Almost all respondents participated in rehabilitation sessions, although slightly fewer adults participated than children and adolescents, 99 vs. 92%, odds ratio 0.21 (0.10–0.45). Physiotherapy sessions, specifically, were attended by 95% of children and adolescents and 87% of adults. Multidisciplinary care decreased sharply from adolescence, 82% in children vs. 64% in adolescents, odds ratio 0.32 (0.19–0.54) and again in adults (41%) vs. adolescents, odds ratio 0.44 (0.27–0.72). Half of children and adolescents reported receiving at least 90 min of PT weekly; the frequency decreased to 42% for young adults (18–25 yo), odds ratio 0.51 (0.27–0.96), and it further decreased to 32% after 25 yo, odds ratio 0.47 (0.25–0.89).

### Motor Rehabilitation Service User Satisfaction and Impact of MR Services

Satisfaction with the current MR program decreased at adolescence, from 58% above the median CSQ-8 score in childhood to 39% in adolescence and adulthood, odds ratio 0.38 (0.25–0.57). Pain management during physiotherapy sessions was reported as strongly satisfying less frequently after 12 yo, 80 vs. 66%, odds ratio 0.52 (0.32–0.85). Goal setting was reported as strongly shared by 56% of children, adolescents and young adults and 52% of adults over 25 yo, odds ratio 0.57 (0.39–0.83). The impact of MR on daily life was reported as positive less frequently in adolescents (31%) than in children, 54%, odds ratio 2.7 (1.6–4.4), or adults, 50%, odds ratio 2.3 (1.4–3.7). The impact of MR on carers was reported as positive less frequently in adolescents and young adults (22%) compared to children, 34%, odds ratio 0.6 (0.38–0.96), and adults over 25 yo, 27%, odds ratio 1.6 (1–2.5).

## Discussion

This study explored the effect of age on a set of MR environmental factors, service use variables and patient outcomes, all of which were reported by people with CP or their main carer. Changes across the lifespan were reported, as hypothesized, for all indicators with, generally, less positive results in adults than children. A more detailed analysis using 4 age categories (2–11, 12–17, 18–24, >25) revealed a wide window of transition between childhood and adulthood that often does not correspond to the pediatric-adult healthcare organization divide. The findings of the study lead to implementing specific actions in motor rehabilitation services in adults, and also in the transition window starting at 12 and up to 24 years of age.

An important result of this study was for accessibility to rehabilitation services: finding an available physiotherapist was reported as highly difficult by almost half of children and an even greater proportion of adolescents and adults. Finding a physiotherapist trained in CP rehabilitation was even more difficult, and this difficulty was increased for adults. The accessibility issues we identified cannot be related to the direct financial cost of MR sessions, as care is fully covered for both children and adults with CP by the healthcare system in France. Instead, accessibility could rather be related to healthcare availability and organization. There was a marked switch in the setting in which rehabilitation was provided. Children and adolescents mainly attended MR in a healthcare organization setting while adults, particularly over 25 yo, mostly had rehabilitation sessions in private outpatient practices. Coincidently, a decrease in the presence of an MR coordinator and in the perceived communication between healthcare professionals was reported at adulthood. Moreover, a distinct lack of multidisciplinary management was observed in adults (41%) and adolescents (61%) compared to children (82%), in contrast with the recent call by the WHO for a stronger multidisciplinary rehabilitation workforce and promotion of the role of allied health professionals in a coordinated strategy aiming at better health outcomes (26). These changes that occurred around 18 years of age suggest that the pediatric healthcare system has well-identified and promoted rehabilitation pathways while the adult system may be less adapted to the needs of people with CP. A failure to provide a seamless transition has been well-described in adulthood (13–15) but has also been described much earlier in the transition from pre-school to school-based services (27), highlighting the need to consider a broader window of transition.

MR service use, especially PT, was reported by almost all participants, even if the rate was slightly lower in adults. This result was expected since, in France, PT is traditionally prescribed, and now recommended (28), as a first-line therapy; it is also consistent with a previous study of adults with CP in a region of France (29). The detailed analysis showed that the amount of weekly physiotherapy provided was lower in young adults than in children and adolescents, and decreased further after the age of 25 years. This finding is in line with data from many countries with different healthcare systems: US (30), Canada (31), UK (32), Australia (33) Singapore (34), as well as in low- and middle-income countries (35). The decrease in rehabilitation service use could be either related to the differences in healthcare provision offered in the French system after 18 years of age, or to a change in specific needs of individuals with CP.

User satisfaction is considered as a key indicator of healthcare service quality (36). Satisfaction with MR was found to be lower in adolescents and adults. Satisfaction with pain management during PT sessions was also lower in adolescents and adults. Lower levels of satisfaction indicate a larger gap between expectations and experiences: in the present study this gap is between perceived rehabilitation needs and care provision. Overall, the results showed a concomitant decrease in satisfaction, amount of rehabilitation, access to rehabilitation services and environmental factors with age. A previous study of satisfaction with MR in CP revealed independent determinants of patient satisfaction (23): higher special needs (pain, impairment severity), lower rehabilitation quality indicators (rehabilitation access, pain management, a lack of shared PT goals and a lack of care coordination), as well as being an adolescent predicted lower levels of satisfaction. Special needs during adolescence were also identified in the current study as the impact of MR on activities of daily living was reported to be less favorable in adolescents and young adults compared to children and adults.

The concurrent decrease in well-organized rehabilitation and patient satisfaction with age reflects an inadequacy between the changing needs of individuals with CP and the healthcare system. This inadequacy occurs roughly in parallel with a marked decrease in the social participation indicator. School attendance was reported below reference population levels for children (90%) and adolescents (77%) in the ESPaCe survey. This has also been well-described in Sweden with a lower rate than typically developing peers (37). Furthermore, the rate of ESPaCe respondents in employment was especially low (18%). “Late adulting” in several social and participation domains has been described in the Netherlands (20), and low levels of employment of people with CP have been reported in several countries (38, 39). These difficulties have been found even in high functioning young adults with CP who have no intellectual disability (40) and in other dimensions of participation like “having a cohabiting partner” or “having a biological descendance” (41). This parallel evolution between systems throughout the wide transition period from childhood to adulthood requires policy makers to develop national strategies that are adapted to the changing needs of individuals with CP in all domains.

### Limits

The study sample represented an estimated 1% of the total population of people with CP living in France. The distribution of CP subtypes was similar to reports in population-based registers, but the proportion with higher levels of gross motor impairment was increased (42, 43). People attending rehabilitation activities were likely overrepresented. Although the sample selection could not guarantee representativeness, the aims of the study were addressed. The age effect estimates were adjusted for gender, CP subtype, GMFCS, single associated impairments (visual, hearing, intelligence and epilepsy), and informant type which is a strong strategy appropriate to the population characteristics. We explored data missingness patterns and concluded that the complete record analysis approach would be appropriate under the assumption that data missingness would not be related to both the main determinant (age) and the study outcomes. We performed sensitivity analyses to assess the effect of rehabilitation non-users at the time of the survey and concluded that a likely underrepresentation of non-users would not bias our results. We also assessed through sensitivity analyses the impact of not including socioeconomic status in the multivariable models due to high data missingness. We concluded that although socioeconomic status may determine rehabilitation access, the estimates of the association between age and outcomes were not impacted by not adjusting for mother's education level. We cannot rule that other participant selection issues, misclassified or unmeasured factors, may have biased the age effect estimates. The questionnaire items selected as indicators did not go through a rigorous validation process but convergent results between indicators were found. These indicators were selected and prioritized by people with CP and advocacy groups and are convergent with the scientific literature, supporting their relevance. More granularity in the age analysis might have provided further information, but the selected transition age groups allowed to analyze the impact of current healthcare features and the study objectives to be addressed.

## Conclusion

This study, which focused on changes in rehabilitation system indicators and patient outcomes with age, brings a new, lifespan vision of how the French healthcare system is perceived and used by people with CP. The results provide grounds for proposing actions at the individual and at the system level: (1) Considering a larger window of transition starting from early adolescence and ending in the late twenties, (2) Developing MR programs that specifically address the needs of adolescents (3) Maintaining a multidisciplinary approach in adulthood (4) Providing access to MR professionals trained in CP at all ages and (5) Promoting pain management and shared goal setting during the MR at all ages but especially in adulthood. Finally, the appropriate management of the needs of people with CP, as reported in this and other studies, is highly challenging for national healthcare systems. This study provides yet further evidence of the need for comprehensive national strategies for the management of individuals with CP that jointly address healthcare, rehabilitation, educational, employment and social support systems.

## Data Availability Statement

The datasets presented in this article are not readily available because consent was not obtained to publish the anonymised data. Requests to access the datasets should be directed to Javier De la Cruz, javier.delacruz@salud.madrid.org.

## Ethics Statement

Ethical review and approval was not required for the study on human participants in accordance with the local legislation and institutional requirements. Written informed consent from the participants' legal guardian/next of kin was not required to participate in this study in accordance with the national legislation and the institutional requirements.

## ESPaCe Committee

E. Bérard, M. Bodoria, J. Boivin, S. Brochard, G. Cornec, J. De la Cruz, I. Desguerre, G. Drewnovski, A. Fontaine, V. Gautheron, G. Geyer, A.-C. Guenier, Y. Le Lay, P. Toullet.

## Author Contributions

All authors listed have made a substantial, direct, and intellectual contribution to the work and approved it for publication.

## Funding

We would like to thank the Fondation Paralysie Cérébrale, Paris, France for its support and funding as well as the co-funders of this work: Envoludia, Fédération Française des Associations des Infirmes Moteurs Cérébraux (FFAIMC), Société d'Etudes et de Soins pour les Enfants Paralysés et Polymalformés (SESEP), Caisse Nationale de solidarité pour l'autonomie (CNSA), KLESIA, L'Oréal Citizen Time.

## Conflict of Interest

The authors declare that the research was conducted in the absence of any commercial or financial relationships that could be construed as a potential conflict of interest.

## Publisher's Note

All claims expressed in this article are solely those of the authors and do not necessarily represent those of their affiliated organizations, or those of the publisher, the editors and the reviewers. Any product that may be evaluated in this article, or claim that may be made by its manufacturer, is not guaranteed or endorsed by the publisher.
